# Prediction of Metabolic Flux Distribution by Flux Sampling: As a Case Study, Acetate Production from Glucose in *Escherichia coli*

**DOI:** 10.3390/bioengineering10060636

**Published:** 2023-05-24

**Authors:** Yuki Kuriya, Masahiro Murata, Masaki Yamamoto, Naoki Watanabe, Michihiro Araki

**Affiliations:** 1 Artificial Intelligence Center for Health and Biomedical Research, National Institute of Biomedical Innovation, Health and Nutrition, 3-17 Senrioka-shinmachi, Settsu 566-0002, Japan; kuriya@nibiohn.go.jp (Y.K.); m.yamamoto@nibiohn.go.jp (M.Y.); n.watanabe@nibiohn.go.jp (N.W.); 2 Graduate School of Science, Technology and Innovation, Kobe University, 1-1 Rokkodai-cho, Nada-ku, Kobe 657-8501, Japan; murata.masahiro.8r@people.kobe-u.ac.jp

**Keywords:** genome-scale metabolic model, flux sampling, flux distribution prediction, important flux extraction

## Abstract

Omics data was acquired, and the development and research of metabolic simulation and analysis methods using them were also actively carried out. However, it was a laborious task to acquire such data each time the medium composition, culture conditions, and target organism changed. Therefore, in this study, we aimed to extract and estimate important variables and necessary numbers for predicting metabolic flux distribution as the state of cell metabolism by flux sampling using a genome-scale metabolic model (GSM) and its analysis. Acetic acid production from glucose in *Escherichia coli* with GSM iJO1366 was used as a case study. Flux sampling obtained by OptGP using 1000 pattern constraints on substrate, product, and growth fluxes produced a wider sample than the default case. The analysis also suggested that the fluxes of iron ions, O_2_, CO_2_, and NH_4_^+^, were important for predicting the metabolic flux distribution. Additionally, the comparison with the literature value of ^13^C-MFA using CO_2_ emission flux as an example of an important flux suggested that the important flux obtained by this method was valid for the prediction of flux distribution. In this way, the method of this research was useful for extracting variables that were important for predicting flux distribution, and as a result, the possibility of contributing to the reduction of measurement variables in experiments was suggested.

## 1. Introduction

Numerous measurement data have been obtained at various layers, such as gene expression, protein, metabolites, and so on. In addition, in metabolic simulations using genome-scale metabolic models (GSMs) for metabolism, there is a growing research effort in developing simulations and analysis methods using omics data and in improving prediction accuracy based on these results [1,2,3,4].

In addition, the mass isotope ^13^C labeling data [5,6,7,8,9,10], metabolome data [1] by LC-MS, and other methods were also used to predict and estimate intracellular metabolic states in previous studies. However, it was still a time-consuming and labor-intensive process to reacquire, simulate, and analyze omics data every time the culture medium composition, culture conditions, or target organisms or strains changed. The more accurate and comprehensive the available data was, the better the prediction accuracy through simulation and analysis, but it was not known how much data was useful for the purpose or how much the prediction accuracy could be improved.

One such previous study was a patent that uses L-lysin production as an example [11]. In this method, the fluxes important for L-lysin production were predicted by (1) elementary mode analysis [12], which was very computationally demanding, and (2) selection of independent metabolic fluxes (called free fluxes) equal to the number of degrees of freedom in the stoichiometric matrix [13], generation of their combinations, and application to metabolic models. However, the GS, a commonly used metabolic model for metabolic simulation, was a very large model in terms of both the number of constituent metabolites and metabolic reactions. Therefore, it was difficult to apply elementary mode analysis due to the limitation of computational costs. Similarly, the method that used the same number of free fluxes as the stoichiometric matrix was difficult to apply in a large-scale model, such as the GSM because the degrees of freedom were very high. Furthermore, it was very difficult to obtain the solution space and its bounds analytically in GSM.

Therefore, in this study, we aimed to propose a search for fluxes and their combinations/numbers that were important for predicting metabolic flux distributions based on simulation using GSM, in view of the labor savings of experimental measurements. Flux sampling is used to obtain the candidate solutions (flux distribution) necessary for this purpose [14]. Flux sampling was a method of sampling a set of possible solutions from the solution space defined by GSM. Algorithms, such as ACHR (the artificially centered hit-and-run) [15], CHRR (the coordinate hit-and-run with rounding) [16], and OptGP (the optimized general parallel) [17], have been implemented, including flux balance analysis (FBA) [18] to find the optimal value for the objective function and flux variability analysis (FVA) [19], which found the range of possible fluxes for an objective function [20,21], which was used to compare and analyze GSMs and metabolic differences due to strain characteristics and conditions, such as correlations between fluxes that could not be determined by these methods alone [20,21]. In this study, OptGP [17], which supports parallelization, was used as the flux sampling algorithm. As a case study, acetic acid production from glucose in *Escherichia coli* was assumed, and iJO1366 [22] was used as the GSM. A comparison of flux sampling results under default conditions [14] with experimental fluxes for substrates, growth, and products suggested that flux sampling under default conditions might not be sufficient to cover the range obtained experimentally for these fluxes. Therefore, the utility of flux sampling was tested by constraining the representative fluxes for substrate, growth, and products to allow for sufficient variation. The flux sampling results were then analyzed to identify the variables important for estimating flux distributions and estimating the number of variables needed. The flux distribution extracted from the flux sampling results using the values of important variables was compared with the ^13^C-MFA results [5] and compared with the flux distribution of the central carbon metabolic pathway. This suggested that the modified flux sampling method used in this study was useful for predicting flux distributions, extracting key variables for this purpose, and estimating the number of necessary variables.

## 2. Materials and Methods

**Figure 1:**
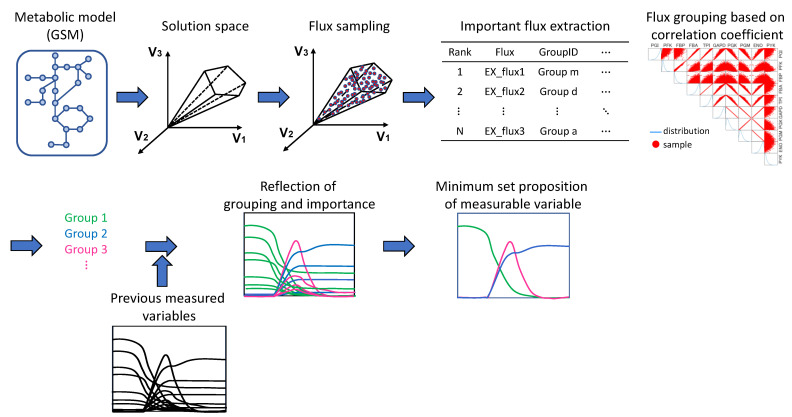
Workflow diagram from the metabolic model (GSM) to the proposal of key measurement variables for predicting metabolic flux distribution. In the flux grouping, the diagonal panels show the distribution of samples, while the other panels show scatter plots of flux pairs in rows and columns. Blue lines indicate sample distributions and red dots indicate samples. The linear scatterplots suggest a high correlation between the two fluxes. The color of the time course data corresponds to the color of the group list, indicating to which group each variable belongs.

The workflow of the simulation and analysis, using the metabolic model as a starting point, is shown in Figure 1.

### 2.1. Metabolic Model

As a test case, a culture of *E. coli* with acetic acid production using glucose as the carbon source was used. The metabolic model used was the *E. coli* GSM iJO1366 [22].

### 2.2. Flux Sampling

ACHR [15], CHRR [16], and OptGP [17], which were commonly used algorithms in flux sampling, were based on a hit-and-run (HR) algorithm. These algorithms performed the next sampling based on the information from the current sampling (current sample, search direction, step size). In the HR algorithm, direction and step size were randomly chosen, iteratively [17]. ACHR algorithm was tailored to sample in the elongated direction of the solution space [15]. With OptGP, more sampling was performed by setting multiple starting points for sampling in ACHR and parallelizing them [17]. On the other hand, it has been suggested that the sampling performance of the HR-based algorithms, including ACHR and OptGP, was greatly affected by the nonuniform shape of solution space [16]. Therefore, CHRR performed faster and more efficient sampling than ACHR by uniformly rounding the solution space [16]. Previous studies have compared the performance of these algorithms, and the results suggested CHRR as the best flux sampling algorithm [14]. However, in CHRR, empirically, when there were multiple fluxes with a very narrow range that could be taken in a large-scale GSM, there were cases where the first rounding of solution space did not go well, and the flux sampling could not proceed. On the other hand, with ACHR and OptGP, flux sampling was possible even with GSMs that did not work well when CHRR was applied (including the *E. coli* GSM iJO1366 used in this study). Therefore, we thought that ACHR and OptGP would be more useful than CHRR in terms of flux sampling in a wider range of GSMs. As a result, we decided to use OptGP [17], as implemented in COBRApy [23], as the flux sampling algorithm.

Flux sampling was compared to the default OptGP, which used the GSM, and OptGP, which ensured sufficient variation in fluxes such as substrates, products, and growth, which were important as phenotypes.

For the default OptGP, flux sampling was performed with the following parameters (thinning = 10,000, sample number = 20,000, and process = 10). For the latter implementation, 1000 patterns of flux value sets were generated using FBA within the range of these three fluxes to ensure sufficient variation to cover the experimentally measured data for the phenotypically important substrate, product, and growth fluxes. First, the specific uptake flux values for the carbon source, glucose, were generated uniformly at random over the predefined range based on experimental data. Next, the specific uptake flux of glucose was fixed at the generated value, and FBA was performed with the objective functions of maximizing and minimizing the specific growth rate for each specific glucose uptake flux, respectively. In this way, the possible range of the specific growth rate was set between the minimum and maximum values of the flux for each glucose uptake condition. The specific growth rate values were randomly selected within each set range. Finally, using the two flux values generated and selected above as constraints, FBA was performed with the objective function of maximizing and minimizing the specific acetic acid production flux, respectively. The possible ranges of specific acetic acid production flux values were similarly set, and those flux values were randomly selected within those ranges. These values were then used as constraints for flux sampling, and sequential flux sampling was performed by OptGP for each constraint (sample number per a flux constraint = 20; other parameters, such as thinning and total number of samples, were the same as the default OptGP shown above).

### 2.3. Verification of the Effect of Using Constraints on Sampling by Dimensional Compression

To verify whether it was important to ensure variations in flux values for substrates, growth, and products, which were important for phenotyping, samples (solution sets) obtained in the same way except with and without the use of constraints were visualized and compared on a two-dimensional plane using multidimensional scaling (MDS) [24].

### 2.4. Search and Evaluation of Fluxes and Combinations of Fluxes Important for Metabolic Flux Distribution Prediction

The search for fluxes important for flux distribution prediction was conducted by (1) selecting any flux and its value, (2) using the selected flux value (±10%) as a query, extracting samples that met the conditions from the generated samples, (3) performing steps (1)–(2) exhaustively for all fluxes and their values, (4) ranking the fluxes based on the average number of samples hit, and (5) considering the fluxes with the highest ranking as important fluxes for the prediction of flux distribution. In the search for important fluxes, since it was difficult to measure intracellular fluxes with high precision, the fluxes taken up from and discharged into the culture medium, which were relatively easy to measure, were targeted here.

On the other hand, the number of fluxes needed to predict flux distribution was estimated from the flux sampling results. Since the number of metabolic fluxes in metabolic models such as the GSM was very large, grouping by correlation among fluxes was used to narrow the number. The samples (flux distributions) obtained by flux sampling were used to determine the correlation coefficient for each flux pair for all fluxes in the metabolic model, and the fluxes were grouped using an absolute value of 0.95 as the threshold value. Using these results, the following procedure was used to estimate the number of fluxes needed to predict the flux distribution. (1) Randomly select one flux from each group obtained from the grouping based on the correlation coefficient. (2) Randomly select one solution from the samples (flux distributions) obtained by flux sampling. (3) Using those values as a query in random order, the solutions were narrowed down from the samples step by step. (4) This was performed for all samples to obtain the minimum number of fluxes required to narrow the solution down to one. (5) (1)–(4) were also performed for 800 different flux combinations (permutations). (6) To estimate the number of fluxes needed to predict the flux distribution, the mean and median of the minimum number of fluxes required to narrow down to one solution in all cases performed were calculated. (7) Fluxes were sorted in descending order by mean and median, and those values were rounded down to the nearest whole number and considered as the number of fluxes needed to predict the flux distribution.

### 2.5. Validation of Important Flux

Since it was difficult to measure many intracellular fluxes experimentally, we used the results of ^13^C-based metabolic flux analysis (MFA) for validation. ^13^C-MFA was the method of choice for detailed inference of intracellular metabolic fluxes in cells or organisms under quasi-steady-state conditions [25]. The carbon source was selected as glucose, and the Zhao and Shimizu [5] literature was used for validation as one of the references from which the acetic acid production flux and the flux values predicted to be important in the previous section could be obtained.

First, values were obtained from the literature for the fluxes considered important for predicting the flux distribution. We selected flux distributions, whose flux values were within ±10% of the literature values, from flux sampling results. The selected flux distributions were compared with the major flux distributions of ^13^C-MFA, and the samples were evaluated for validity by the mean absolute percent error of those flux values.

### 2.6. Computer Code and Software

In this research, all simulation and calculation were run on a server with CPU Xeon Gold 6136 (3GHz) ×2, and Memory 512GB. Also, COBRA Toolbox v3,33 (https://github.com/opencobra/cobratoolbox.git (accessed on 4 October 2021)), MATLAB© 2018b (The MathWorks Inc., Natick, MA, USA), COBRApy v0.22.1 (https://github.com/opencobra/cobrapy (accessed on 19 January 2022)), GLPK v5.0 (Gnu Linear Programming Kit) were used. Scripts (computer codes) were used for flux balance analysis, flux variability analysis, production envelope, flux sampling, sampling analysis, and are available at github (https://github.com/yukuriya3/fluxsampling_for_pred (accessed on 23 April 2023)).

## 3. Results

### 3.1. Creating Constraints for Flux Sampling

**Figure 2:**
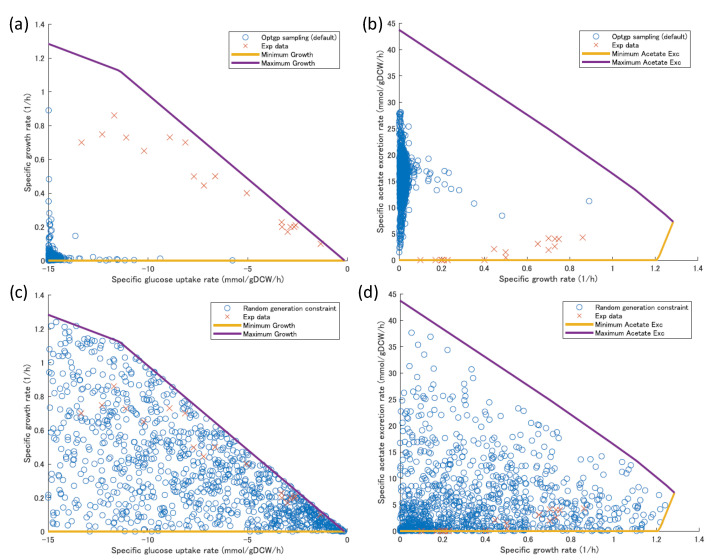
The possible ranges of the three fluxes for substrate, product, and growth and the 1000 patterns of seed (constraints) generated for the samples and flux sampling generated by the default OptGP. The metabolic model used was iJO1366 for *E. coli*. The panel (**a**,**b**) shows the results obtained by flux sampling with OptGP under default conditions (open circles) and the samples obtained by the experiment (cross), and the panel (**c**,**d**) shows the 1000 patterns of seeds (constraints) for the three fluxes generated for the flux sampling. The panel (**a**,**c**) shows the specific glucose uptake flux and the specific growth rate, and the panel (**b**,**d**) shows the specific growth rate and the specific acetic acid production rate. The solid lines and axes indicate the range of possible fluxes, the open circles indicated the seeds (constraints) generated for flux sampling, and the crosses indicate samples obtained from the literatures of the experiment.

When flux sampling was performed with OptGP under default conditions, the variation in the sample values obtained was biased toward a narrow range of fluxes for the phenotypically important substrates, products, and growth, and did not cover the sample values obtained by the experiment (Figure 2a,b). Therefore, 1000 patterns of flux values were generated by the method of the M and M section to provide sufficient variation in those three flux values. The obtained values for the three fluxes were widely distributed within the range of possible values, indicating that they covered the experimentally obtained flux values (Figure 2c,d). The flux values thus generated were used as a seed (constraint) to perform flux sampling.

### 3.2. Flux Sampling

**Figure 3:**
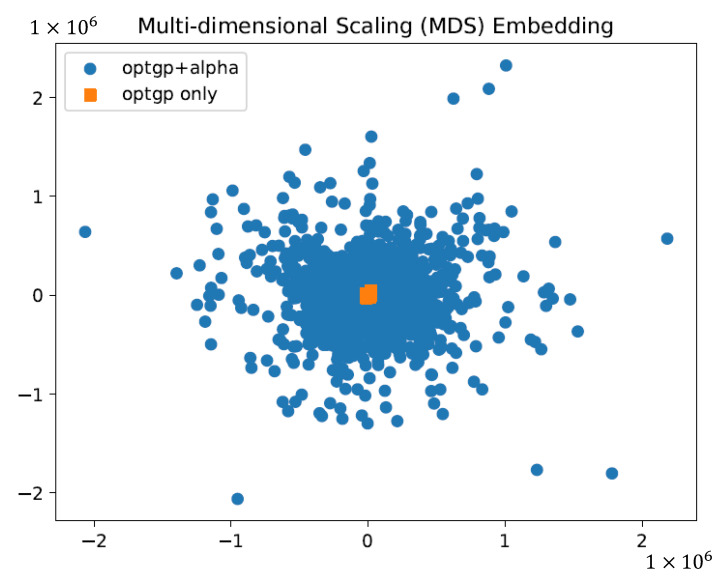
Comparison of sampling results of OptGP (optgp and alpha) using sequential constraint conditions and OptGP (optgp only) using default conditions on *E. coli* iJO1366. Blue circles and orange squares indicate the results of OptGP with sequential use of 1000 constraint patterns and OptGP with default conditions, respectively.

Flux sampling was sequentially performed using OptGP with the 1000-pattern constraint conditions generated above to ensure variation in the three phenotypically important fluxes. To investigate the effect of using the constraints generated above, the specific uptake flux of glucose as a carbon source was normalized to 100, and the results of sampling by OptGP using the constraints and the default conditions were compared by visualizing them on a two-dimensional plane using dimensional compression by MDS (Figure 3). MDS is a method of placing similar objects closer to each other and different objects farther apart. Therefore, the results suggested that the OptGP sample using sequential use of the generated constraints, which plotted results over a wider range, was a more diverse sample.

### 3.3. Exploration and Evaluation of Fluxes and Combinations of Fluxes Important for Flux Distribution Prediction

Arbitrary fluxes and their values were used to select fluxes that are important for flux distribution prediction. The resulting top fluxes selected were fluxes EX_fe2_e, EX_fe3_e, EX_co2_e, EX_o2_e, and EX_nh4 _e (Table 1).

The GSM iJO1366 model for *E. coli* yielded 457 groups by grouping, using the absolute value of the correlation coefficient. The fluxes constituting each group are shown in Supplementary Materials Table S1. We used these to investigate the mean and median of the minimum number of fluxes required to narrow down any one solution from the samples (flux distributions) for the order of application of 800 different group fluxes, and found them to be approximately 7.20 and 6.0, respectively.

### 3.4. Validation of Important Flux

The results of metabolic flux analysis with ^13^C obtained from the literature [5] were used to validate the fluxes important for the prediction of the flux distribution obtained from the flux sampling results.

**Figure 4:**
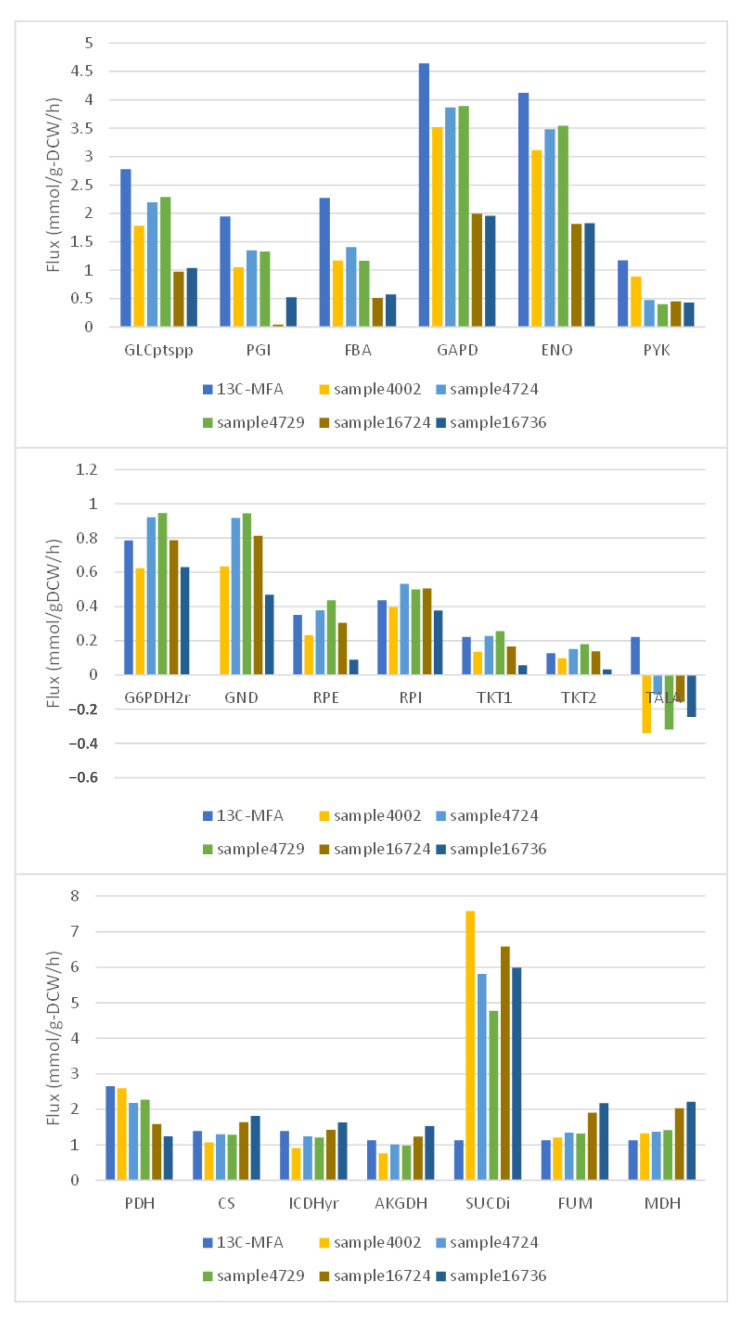
Comparison of flux values for the central carbon metabolic system obtained by flux sampling and ^13^C-MFA. From the top, the figure compares the fluxes of the glycolytic system, the pentose phosphate pathway, and the TCA cycle. Literature values for the ^13^C-MFA of the pentose phosphate pathway flux GND were missing because no explicit values were found in the literature.

Among the fluxes selected as important fluxes as described above, CO_2_ emissions were selected as a flux that could be obtained from ^13^C-MFA results, and its value was extracted. Next, samples were extracted from the flux sampling results (10 samples) that fell within a range of ±10% of that flux value with an assumed measurement error. Then, from these samples, we selected the five solutions with the lowest mean absolute percentage error (MAPE) (Table 2). The obtained solution candidates were compared with the flux values of the central carbon metabolism (glycolytic pathway, pentose phosphate pathway, and TCA cycle) by ^13^C-MFA (Figure 4). In Figure 4, the leftmost bar shows the results from ^13^C-MFA and the other five bars show the results of samples extracted by the above procedure from the flux sampling results. The results obtained from flux sampling were smaller than those from ^13^C-MFA for glucose uptake flux by PTS (GLCptspp) and glycolytic start flux (PGI). Conversely, the results obtained from flux sampling were higher than those from ^13^C-MFA for the starting flux of PPP (G6PDH2r). On the other hand, for transaldolase of PPP, the sign of the flux values obtained from ^13^C-MFA and flux sampling were different. Furthermore, the flux value for succinate dehydrogenase in the TCA cycle was much higher for the results obtained from flux sampling than for ^13^C-MFA.

## 4. Discussion

Flux sampling was performed using acetic acid production from glucose using *E. coli* GSM iJO1366 as a case study.

For flux sampling, 1000 patterns of constraints were generated that ensured sufficient variability for phenotypically important substrate, product, and growth-related fluxes and were used for sampling by OptGP. Normalization of the results by specific glucose uptake flux and visualization by MDS suggested an improvement to obtain a wider and more diverse sample (Figure 3). On the other hand, it was difficult to accurately estimate the volume of the solution space or the volume of the sampled space [26,27]. Therefore, it was unclear how much of the entire solution space was sampled and what was needed to improve the sampling.

Using the flux values in the sample obtained by the modified OptGP to search for and extract solutions from the entire sample allowed us to evaluate and extract fluxes (variables) that were important for estimating the flux distribution based on the number of solutions obtained. Apart from the fluxes of water, protons, and the fluxes of glucose, growth, and acetic acid, which were used as constraints, the fluxes of iron, oxygen, carbon dioxide, ammonia, and inorganic phosphate were the most important fluxes. For these fluxes, they were relatively easy to measure and were generally included in the culture on minimal media. Therefore, although the study was conducted only for *E. coli*, if these fluxes could also be obtained from flux sampling results for a wide range of micro-organisms, it was expected that they might be fluxes that should be commonly measured during the culture of many micro-organisms. The results of this study also suggested that fluxes related to iron would be particularly useful in predicting flux distribution. Biologically, iron uptake affected *E. coli* growth [28,29], while iron metabolism and homeostasis were strictly regulated [30]. *E. coli* GSM iJO1366 contained iron-related fluxes, such as uptake and efflux of divalent and trivalent iron ions, biomass synthesis, iron-sulfur clusters, and multiple redox reactions. The fluxes related to iron were found to be important fluxes in this study, partly because the possible range of these fluxes was relatively larger than the other fluxes. However, whether this was due to the formation of futile cycles in the GSM or a lack of regulatory information, it was considered to require a more detailed investigation. 

On the other hand, as mentioned in Section 2.4, to estimate the number of measurement variables needed to approximately predict the metabolic flux distribution indicative of the metabolic state of the cell, we investigated the minimum number of fluxes needed to narrow the solution to one within the obtained sample. The results suggested that seven or eight fluxes were generally sufficient to estimate the flux distribution when combining flux values from different groups, based on groupings conducted based on an absolute value of correlation coefficients. Since these fluxes included data obtained by the analysis of the culture medium supernatant and gas analyses, combining variables that were relatively easy to measure would be sufficient to estimate the flux distribution.

Due to the difficulty of measuring flux values, we used the results of ^13^C-MFA to validate the fluxes that were important in predicting the obtained flux distributions. Flux distributions with values close to those from the flux sampling results were extracted. The obtained flux distributions were then compared with the ^13^C-MFA results. The resulting flux distribution had a mean absolute percentage error of approximately 54% from the closest flux distribution. This was because the fluxes of transaldolase in the pentose phosphate pathway and of succinate dehydrogenase in the TCA cycle differed significantly from the ^13^C-MFA results. In particular, the fluxes of succinate dehydrogenase, which differed greatly between the two, were thought to form futile cycles with other fluxes in GSM. Therefore, if the fluxes of succinate dehydrogenase were excluded, the difference between ^13^C-MFA and the results extracted from the flux sampling would be greatly reduced. Considering the difference between ^13^C-MFA in the glucose uptake flux, which was the starting point of carbon metabolism, and those in the flux sampling results, it was thought that the two central carbon metabolism fluxes were quite close. Therefore, this result suggested that the fluxes important in predicting the flux distribution obtained from the flux sampling results were valid.

OptGP flux sampling was performed under different constraints with sufficient variation to include experimentally obtained flux values for phenotypically important substrate, product, and growth fluxes. As a result, flux sampling could be carried out in a wider range than OptGP without additional constraints. In addition, although this study was limited to acetic acid production from glucose in *E. coli*, these results suggested that the analysis of the result obtained from flux sampling by OptGP with additional constraints could provide information about the important variables, their numbers, and combinations to predict flux distribution. This meant that, instead of performing the time-consuming and labor-intensive task of obtaining tens or hundreds of items of omics data every time the host, culture condition, or target product changed, we could predict and narrow down the variables to be measured and their numbers in advance based on simulation using GSM. In addition, by reducing the number of variables to be measured, a more accurate measurement of them could be expected. Thus, the method presented in this study was considered very useful in reducing the cost of experimental work on a simulation basis.

In the future, we will plan to combine the method presented in this study with reduced cost, which corresponds to the sensitivity analysis of FBA using GSM, to improve the method and to verify its versatility in GSMs of various micro-organisms, and investigate flux sampling under nutrient-rich culture conditions with a larger solution space.

## Figures and Tables

**Table 1:** Important fluxes for prediction of metabolic flux distribution.

Rank	Flux Name	Group ID	Flux ID	Sol. Num. (Ave.) ^1^	Sol. Num. (Med.) ^2^
1	EX_fe2_e	11	127	1.3685	1
2	EX_fe3_e	11	128	1.3763	1
3	EX_h_e	11	185	1.3768	1
4	EX_h2o_e	11	187	1.742	2
5	EX_o2_e	11	252	2.4766	2
6	EX_co2_e	30	85	11.355	10
7	EX_nh4_e	2	244	33.095	32
8	EX_glc__D_e	457	164	40.319	40
9	EX_ac_e	452	36	49.47	40
10	EX_pi_e	2	263	364.97	337

^1^ Sol. Num. (ave.): averaged solution number. ^2^ Sol. Num. (med.): median of solution number.

**Table 2:** Top 5 samples with the smallest mean absolute percentage error relative to ^13^C-MFA.

Sample ID	Sample4002	Sample4724	Sample4729	Sample16724	Sample16736
MAPE ^1^	83.8828	54.1644	57.1455	77.5504	88.2746

^1^ MAPE: mean absolute percentage error.

## Data Availability

The data presented in this study are openly available at reference number [5,6,7,8,9,10] and generated with scripts and data of github [https://github.com/yukuriya3/fluxsampling_for_pred (accessed on 23 April 2023)].

## Supplementary Materials

The following supporting information can be downloaded at: https://www.mdpi.com/article/10.3390/bioengineering10060636/s1, Table S1: List of fluxes grouped based on correlation coefficients.
