# Supplementary material for: Prediction of Metabolic Flux Distribution by Flux Sampling: As a Case Study, Acetate Production from Glucose in Escherichia coli
Source: Bioengineering (Basel). 2023 May 24;10(6):636. doi: 10.3390/bioengineering10060636 (PMC10295088; doi:10.3390/bioengineering10060636)
Supplement: Supplementary file 1 [file bioengineering-10-00636-s001.zip › bioengineering-2383591-supplementary.pdf]

Supplementary Table S1 List of fluxes grouped based on correlation coefficients

Group	Flux					
1	'DM_4crsol_	'DM_amob_	'BIOMASS_	'EX_ca2_e'	'EX_cl_e'	'EX_cobalt2	'EX_cu2_e'
	'EX_k_e'	'EX_meoh_	'EX_mg2_e'	'EX_mn2_e'	'EX_mobd_	'EX_ni2_e'	'EX_zn2_e'
	'ACACT1r'	'ACACT2r'	'ACOAD1f'	'ACOAD2f'	'ADCL'	'ADCS'	'AMAOTr'
	'AMPMS2'	'AOXSr2'	'APRAUR'	'ASP1DC'	'ASPO6'	'BMOCOS'	'BMOGDS1'
	'BMOGDS2'	'BTS5'	'CA2tex'	'CDPMEK'	'CHRPL'	'CLt3_2pp'	'CLtex'
	'COBALT2t	'CPMPS'	'CU2tex'	'DB4PS'	'DBTS'	'DHFS'	'DHNPA2r'
	'DHPPDA2'	'DHPS2'	'DMATT'	'DNMPPA'	'DNTPPA'	'DPCOAK'	'DPR'
	'DXPRIi'	'DXPS'	'E4PD'	'ECOAH1'	'ECOAH2'	'EGMEACP	'EPMEACPR'
	'FMNAT'	'GCALDD'	'GRTT'	'GTPCI'	'GTPCII2'	'HACD1'	'HACD2'
	'HBZOPT'	'HPPK2'	'I2FE2SR'	'I2FE2SS'	'I2FE2SS2'	'I2FE2ST'	'I4FE4SR'
	'I4FE4ST'	'ICYSDS'	'IPDPS'	'IPMD'	'IPPMIa'	'IPPMIb'	'IPPS'
	'Ktex'	'LEUTAi'	'MALCOAM	'MOBDtex'	'MOCOS'	'MOHMT'	'MPTAT'
	'MPTS'	'MPTSS'	'NI2tex'	'NNDPR'	'OCTDPS'	'OGMEACP	'OGMEACPR'
	'OGMEACP	'OHPBAT'	'OMCDC'	'OPHBDC'	'MECDPDH	'MECDPS'	'MEOHtex'
	'MEOHtrpp'	'MEPCT'	'MG2tex'	'MNtex'	'MOADSUx'	'MOBDabcp	'OPHHX'
	'OPMEACP	'OPMEACP	'OPMEACP	'PANTS'	'PDX5PS'	'PERD'	'PMDPHT'
	'PMEACPE'	'PNTK'	'PPCDC'	'PPNCL2'	'PPND'	'PTPATi'	'QULNS'
	'RBFK'	'RBFSa'	'RBFSb'	'SHCHD2'	'SHCHF'	'THZPSN3'	'TMPPP'
	'TYRL'	'TYRTA'	'UDCPDPS'	'UPP3MT'	'Zn2tex'		
2	'EX_nh4_e'	'EX_pi_e'	'ACHBS'	'ACLS'	'ATPPRT'	'CHORM'	'CHORS'
	'CYSTL'	'DAPDC'	'DAPE'	'DDPA'	'DHAD1'	'DHAD2'	'DHDPRy'
	'DHDPS'	'DHQS'	'DHQTi'	'HISTD'	'HISTP'	'HSST'	'HSTPT'
	'IG3PS'	'IGPDH'	'ILETA'	'KARA1'	'KARA2'	'METS'	'MTHFR2'
	'NH4tex'	'NH4tpp'	'PHETA1'	'PItex'	'PPNDH'	'PRAMPC'	'PRATPP'
	'PRMICI'	'PSCVT'	'SDPDS'	'SDPTA'	'SHK3Dr'	'SHKK'	'SHSL1'
	'THDPS'	'THRD_L'					
3	'AGMHE'	'COLIPAabc	'GALT1'	'GLCTR1'	'GLCTR2'	'GLCTR3'	'GMHEPAT'
	'GMHEPK'	'GMHEPPA'	'HEPK1'	'HEPK2'	'HEPT1'	'HEPT2'	'HEPT3'
	'HEPT4'	'MOAT3C'	'RHAT1'	'S7PI'	'TDPDRE'	'TDPDRR'	
4	'EX_eca4co	'AADDGT'	'ACGAMT'	'ACMAMUT	'ECA4COLI	'ECA4OALp	'ECAP1pp'
	'ECAP2pp'	'ECAP3pp'	'ECAtpp'	'G1PTT'	'TDPADGA	'TDPAGTA'	'TDPGDH'
	'UACMAMO	'UAG2E'	'UDCPDPp	'UDCPPtppi'			
5	'ACGAMK'	'ACM6PH'	'AGDC'	'AGMH'	'ANHMK'	'DALAt2pp'	'G1PACT'
	'MPTG'	'PAPPT3'	'PGAMT'	'UAGCVT'	'UAGDP'	'UAGPT3'	'UAPGR'
	'UDCPDP'	'UGMDDS'	'UM3PL'				
6	'A5PISO'	'K2L4Aabcp	'K2L4Aabct	'KDOCT2'	'KDOPP'	'KDOPS'	'LPADSS'
	'MOAT'	'MOAT2'	'TDSK'	'U23GAAT'	'UAGAAT'	'UHGADA'	'USHD'

7	'EX_h2o2_e	'3OXCOAT'	'ALDD19xr'	'DHACOAH'	'H2O2tex'	'HADPCOAI	'OXCOAHDH'
	'OXDHCOA	'PACALDt2r	'PACCOAE'	'PACCOAL'	'PEAMNOp	'PEAMNtex'	'REPHACCOAI'
8	'ACACT3r'	'ACACT4r'	'ACACT5r'	'ACOAD3f'	'ACOAD4f'	'ACOAD5f'	'ECOAH3'
	'ECOAH4'	'ECOAH5'	'HACD3'	'HACD4'	'HACD5'		
9	'3HAD40'	'3HAD60'	'3HAD80'	'3OAR40'	'3OAR60'	'3OAR80'	'3OAS60'
	'3OAS80'	'EAR40x'	'EAR80x'	'KAS14'			
10	'EX_pheme_	'FCLT'	'G1SAT'	'GLUTRR'	'GLUTRS'	'HMBS'	'PHEMEabcpp'
	'PHEMEtiex	'PPBNGS'	'UPP3S'	'UPPDC1'			
11	'EX_fe2_e'	'EX_fe3_e'	'EX_h_e'	'EX_h2o_e'	'EX_o2_e'	'FE2tex'	'FE3tex'
	'FEROpp'	'H2Otex'	'Htex'	'O2tex'			
12	'ADSL2r'	'AICART'	'AIRC2'	'AIRC3'	'GLUPRT'	'IMPC'	'PRAGSr'
	'PRAIS'	'PRASCSi'	'PRFGS'				
13	'EX_acolipa	'ACOLIPAat	'LA4NTpp'	'UDPGD'	'UDPGDC'	'UDPKAAT'	'ULA4NFT'
	'ULA4Ntppi'	'UPLA4FNF	'UPLA4FNT'				
14	'EX_udpgal_	'GAL1PPpp'	'GALKr'	'GALt2pp'	'NTD2pp'	'UDPG4E'	'UDPGALPpp'
	'UDPGALte	'UGLT'					
15	'EX_3hpp_e	'3AMACHY	'3HPPtex'	'3HPPtpp'	'CBMD'	'MSAR'	'POAACR'
	'PYROX'	'URACPAH'					
16	'EX_5mtr_e	'EX_spmd_	'5MTRtex'	'5MTRtpp'	'ADMDC'	'MTAN'	'SPMDtex'
	'SPMS'						
17	'HPYRI'	'HPYRRx'	'TRSARr'	'URAt2pp_c	'URAt2pp_c	'URIt2pp_cc	'URIt2pp_copy2'
18	'DHNAOT4'	'DHNCOAS'	'DHNCOAT'	'SEPHCHC	'SHCHCS3'	'SUCBZL'	'SUCBZS'
19	'ALAGLUE'	'GLUR'	'LADGMDH'	'LALGP'	'UAAGDS'	'UAMAGS'	'UAMAS'
20	'ACONTa'	'ACONTb'	'AKGDH'	'CS'	'FUM'	'ICDHyr'	'MDH'
21	'3HAD121'	'3HAD141'	'3OAR121'	'3OAR141'	'3OAS121'	'3OAS141'	'T2DECAI'
22	'EX_so4_e'	'ADSK'	'BPNT'	'SADT2'	'SO4t2pp'	'SO4tex'	'SULR'
23	'EX_n2o_e'	'EX_no_e'	'FESD2s'	'N2Otex'	'N2Otpp'	'NOtex'	'NOtpp'
24	'DM_mththf_	'AHCYSNS'	'DHPTDCs2	'HCYSMT'	'METAT'	'MTHTHFSs	'RHCCE'
25	'ENO'	'GAPD'	'GLCptspp'	'PGK'	'PGM'	'TPI'	
26	'MCITD'	'MCITL2'	'MCITS'	'MICITDr'	'MMCD'	'MMM'	
27	'ACBIPGT'	'ADOCBIK'	'CBIAT'	'CBIuabcpp'	'NNDMBRT	'RZ5PP'	
28	'EX_sel_e'	'EX_slnt_e'	'SELtex'	'SELtpp'	'SLNTtex'	'SLNTtpp'	
29	'EX_fuc__L_	'FCI'	'FCLK'	'FCLPA'	'FUCtex'	'FUCtpp'	
30	'EX_co2_e'	'ATPS4rpp'	'CO2tex'	'CO2tpp'	'CYTBO3_4	'O2tpp'	
31	'S2FE2SS'	'S2FE2SS2'	'S4FE4SR'	'S4FE4ST'	'SCYSDS'		
32	'O16AP1pp'	'O16AP2pp'	'O16AP3pp'	'O16AUNDt	'O16GLCT2'		
33	'MCTP2App	'MDDCP2pp	'MDDEP4pp	'MLTGY4pp	'MPTG2'		
34	'GLCURt2rp	GUI1'	'GUR1PPpp	'UDPGLCUI	'UGLCURPpp'		
35	'FEOXAMU	'FEOXAMU	'FEOXAMat	'FEOXAMex	'FEOXAMtonex'		

36	'FEENTERa	'FEENTERe	'FEENTERt	'FEENTERt	'FEENTERtpp'
37	'FECRMUte	'FECRMUtp	'FECRMabc	'FECRMexs	'FECRMtonex'
38	'FE3HOXUt	'FE3HOXUt	'FE3HOXab	'FE3HOXex	'FE3HOXtonex'
39	'DMQMT'	'OHPHM'	'OMBZLM'	'OMMBLHX'	'OMPHHX'
40	'DHBS'	'DHBSH'	'ENTCS'	'ENTERES'	'SERASr'
41	'CPGNUtex'	'CPGNUtpp'	'CPGNabcp	'CPGNexs'	'CPGNtonex'
42	'AST'	'SADH'	'SGDS'	'SGSAD'	'SOTA'
43	'ASAD'	'ASPK'	'HSDy'	'HSK'	'THRS'
44	'ARBTNabc	'ARBTNexs'	'ARBTNtex'	'ARBTNtone	'ARBTNtpp'
45	'ANPRT'	'ANS'	'IGPS'	'PRAIi'	'TRPS3'
46	'AMPTASE(	'CGLYabcp	'GLUCYS'	'GTHRDHp	'GTHS'
47	'ALLTAMH'	'ALLTN'	'UGLYCH'	'URIC'	'XAND'
48	'ALAt2pp_c	'ASPt2pp_c	'ASPt2pp_c	'CA2t3pp'	'CAt6pp'
49	'ACGK'	'ACGS'	'ACODA'	'ACOTA'	'AGPR'
50	'EX_lipa_co	'CLIPAabcte	'EDTXS3'	'EDTXS4'	'LIPACabcpp'
51	'EX_fald_e'	'EX_mso3_e	'FDMO2'	'MSO3abcp	'MSO3tex'
52	'EX_14gluca	'14GLUCAN	'AMALT4'	'MLTG1'	'MLTG2'
53	'PPM'	'PRPPS'	'R15BPK'	'R1PK'	
54	'ICHORS_c	'ICHORS_c	'INDOLEt2p	'INDOLEt2rpp'	
55	'GGGABAD	'GGGABAH	'GGPTRCO	'GGPTRCS'	
56	'G3PIabcpp'	'GPDDA5'	'GPDDA5pp	'INOSTt4pp'	
57	'DHCINDO'	'HCINNMt2r	'HCINNMte	'HKNTDH'	
58	'ASPCT'	'DHORTS'	'OMPDC'	'ORPT'	
59	'ALDD4'	'BUTSO3ab	'BUTSO3te	'FDMO4'	
60	'ADOCBLab	'ADOCBLto	'CBL1abcpp	'CBLAT'	
61	'3HAD181'	'3OAR181'	'3OAS181'	'CTECOAI8'	
62	'3HAD180'	'3OAR180'	'3OAS180'	'ACOAD8f'	
63	'2AGPG180	'2AGPGAT1	'PG180abcp	'PLIPA1G180pp'	
64	'2AGPG160	'2AGPGAT1	'PG160abcp	'PLIPA1G160pp'	
65	'2AGPG120	'2AGPGAT1	'PG120abcp	'PLIPA1G120pp'	
66	'2AGPE181	'2AGPEAT1	'PE181abcp	'PLIPA1E181pp'	
67	'2AGPE180	'2AGPEAT1	'PE180abcp	'PLIPA1E180pp'	
68	'2AGPE160	'2AGPEAT1	'PE160abcp	'PLIPA1E160pp'	
69	'12DGR181	'DAGK181'	'PA181abcp	'PAPA181pp'	
70	'EX_urea_e'	'AGMT'	'UREAtex'	'UREAtpp'	
71	'EX_tma_e'	'TMAOR1pp	'TMAOtex'	'TMAtex'	
72	'EX_thym_e	'THYMt3pp'	'THYMtex'	'TMDPP'	
73	'EX_quin_e'	'QUIN2tex'	'QUIN2tpp'	'QUINDH'	
74	'EX_idon__l	'5DGLCNR'	'IDONt2rpp'	'IDONtex'	

75	'EX_glyald_(	'ALCD19'	'GLYALDtex	'GLYALDtpp'
76	'EX_etoh_e'	'ALCD2x'	'ETOHtex'	'ETOHtrpp'
77	'EX_acgal1p	'ACGAL1Pte	'UACGALPp	'UDPACGALtex'
78	'EX_acgal_e	'ACGAL1PP	'ACGALtex'	'G1Ptex'
79	'EX_15dap_	'CADVtpp'	'DAPtex'	'LYSDC'
80	'EX_12ppd_	'12PPDStex	'12PPDStpp	'LCARS'
81	'DM_aacald_	'EX_taur_e'	'TAURabcpp	'TAURtex'
82	'RPE'	'TKT1'	'TKT2'	
83	'PGCD'	'PSERT'	'PSP_L'	
84	'NADS1'	'NMNDA'	'NNATr'	
85	'MLDCP3Ap	'MLDEP1pp	'MLTGY3pp'	
86	'LPLIPAL2E	'PSD180'	'PSSA180'	
87	'LPLIPAL2E	'PSD161'	'PSSA161'	
88	'LPLIPAL2E	'PSD120'	'PSSA120'	
89	'GLCDpp'	'GLCNt2rpp	'GNK'	
90	'GHMT2r'	'MTHFC'	'MTHFD'	
91	'GALCTLO'	'GALCTNLt2	'GALCTNLtex'	
92	'G6PDH2r'	'GND'	'PGL'	
93	'G1PPpp'	'UDPGPpp'	'UDPGtex'	
94	'FE3Ri'	'FESD1s'	'FESR'	
95	'FDMO6'	'SULFACab	'SULFACtex'	
96	'FDMO'	'ISETACabc	'ISETACtex'	
97	'ETHSO3ab	'ETHSO3tex	'FDMO3'	
98	'EDTXS1'	'EDTXS2'	'LIPAabcpp'	
99	'DTMPK'	'NDPK4'	'NTPP7'	
100	'DSERDHr'	'LSERDHr'	'SERD_D'	
101	'DMSOR2'	'DMSOtex'	'DMSOtpp'	
102	'DATPHs'	'NTD12'	'NTPP10'	
103	'DADK'	'NTD6'	'PUNP2'	
104	'CRNDt2rpp	'CRNt2rpp'	'CRNt8pp'	
105	'ARGSL'	'ARGSS'	'OCBT'	
106	'ARGORNt7	'ARGt3pp'	'PTRCORNt7pp'	
107	'ARAI'	'RBK_L1'	'RBP4E'	
108	'ALATA_L'	'VALTA'	'VPAMTr'	
109	'ACNAMt2p	'AMANAPEr	'AMANK'	
110	'ACKr'	'ACS'	'PTAr'	
111	'ACCOAC'	'HCO3E'	'MCOATA'	
112	'ACALD'	'DRPA'	'PPM2'	
113	'ACACT8r'	'ECOAH8'	'HACD8'	

114	'ACACT7r'	'ECOAH7'	'HACD7'	
115	'ACACT6r'	'ECOAH6'	'HACD6'	
116	'ABUTt2pp'	'GLUABUTt'	'GLUt2rpp'	
117	'3HAD161'	'3OAR161'	'3OAS161'	
118	'3HAD160'	'3OAR160'	'3OAS160'	
119	'3HAD140'	'3OAR140'	'3OAS140'	
120	'3HAD120'	'3OAR120'	'3OAS120'	
121	'3HAD100'	'3OAR100'	'3OAS100'	
122	'2AGPG181	'2AGPGAT1	'PLIPA1G181pp'	
123	'2AGPG161	'2AGPGAT1	'PLIPA1G161pp'	
124	'2AGPG141	'2AGPGAT1	'PLIPA1G141pp'	
125	'2AGPG140	'2AGPGAT1	'PLIPA1G140pp'	
126	'2AGPE161	'PE161abcp	'PLIPA1E161pp'	
127	'2AGPE141	'PE141abcp	'PLIPA1E141pp'	
128	'2AGPE140	'PE140abcp	'PLIPA1E140pp'	
129	'2AGPE120	'PE120abcp	'PLIPA1E120pp'	
130	'2AGPA181	'LPLIPAL2A	'PLIPA1A181pp'	
131	'2AGPA180	'LPLIPAL2A	'PLIPA1A180pp'	
132	'2AGPA161	'LPLIPAL2A	'PLIPA1A161pp'	
133	'2AGPA160	'LPLIPAL2A	'PLIPA1A160pp'	
134	'2AGPA141	'LPLIPAL2A	'PLIPA1A141pp'	
135	'2AGPA140	'LPLIPAL2A	'PLIPA1A140pp'	
136	'2AGPA120	'LPLIPAL2A	'PLIPA1A120pp'	
137	'12DGR161	'PA161abcp	'PAPA161pp'	
138	'EX_xtsn_e'	'XTSNt2rpp'	'XTSNtex'	
139	'EX_tyr__L_	'TYRt2rpp'	'TYRtex'	
140	'EX_trp__L_	'TRPt2rpp'	'TRPtex'	
141	'EX_thm_e'	'THMabcpp'	'THMtex'	
142	'EX_pyr_e'	'PYRt2rpp'	'PYRtex'	
143	'EX_pydam_	'PYDAMtex'	'PYDAMtpp'	
144	'EX_pnto__I	'PNTOt4pp'	'PNTOtex'	
145	'EX_phe__L	'PHEt2rpp'	'PHEtex'	
146	'EX_o16a4colipa_e'		'O16A4COL	'O16A4Lpp'
147	'EX_lac__L_	'L_LACt2rpp	'L_LACtex'	
148	'EX_lac__D_	'D_LACt2pp	'D_LACtex'	
149	'EX_hxan_e	'HYXNtex'	'HYXNtpp'	
150	'EX_hom__I	'HOMt2pp'	'HOMtex'	
151	'EX_h2s_e'	'H2St1pp'	'H2Stex'	
152	'EX_h2_e'	'H2tex'	'H2tpp'	

153	'EX_gthox_e	'DSBCGT'	'GTHOXtex'
154	'EX_glyc__F	'GLYCAt2rp	'GLYCAtex'
155	'EX_glyb_e'	'GLYBt2pp'	'GLYBtex'
156	'EX_dms_e'	'DMSOR1pp	'DMStex'
157	'EX_dha_e'	'DHAtex'	'DHAtpp'
158	'EX_colipap_	'COLIPAKpp	'COLIPAPabctex'
159	'EX_btn_e'	'BTNt2ipp'	'BTNtex'
160	'EX_alltn_e'	'ALLTNt2rpp	'ALLTNtex'
161	'EX_akg_e'	'AKGt2rpp'	'AKGtex'
162	'EX_ade_e'	'ADEt2rpp'	'ADEtex'
163	'EX_acser_e	'ACSERtex'	'ACSERtpp'
164	'EX_acald_e	'ACALDtex'	'ACALDtpp'
165	'EX_LalaLgl	'LALALGLU	'LALALGLUtpp'
166	'EX_5dglcn_	'5DGLCNt2r	'5DGLCNtex'
167	'EX_12ppd_	'12PPDRtex	'12PPDRtpp'
168	'DM_oxam_	'OXAMTC'	'URDGLYCD'
169	'DM_5drib_c	'5DOAN'	'CPPPGO2'
170	'ZN2t3pp'	'ZN2tpp'	
171	'XANt2pp'	'XANtpp'	
172	'VALabcpp'	'VALt2rpp'	
173	'UM4PCP'	'UM4PL'	
174	'TTRCYCtex	'TTRCYCtpp'	
175	'TRPAS2'	'TRPS2'	
176	'TRE6PP'	'TREH'	
177	'THRt2rpp'	'THRt4pp'	
178	'THMDt2pp_	'THMDt2pp_copy2'	
179	'TDP'	'TMPK'	
180	'SPMDabcp	'SPMDt3pp'	
181	'SERt2rpp'	'SERt4pp'	
182	'S2FE2SR'	'S2FE2ST'	
183	'PUNP4'	'RNTR2c2'	
184	'RMI'	'RMNtpp'	
185	'RFAMPtex'	'RFAMPtpp'	
186	'R5PPpp'	'R5Ptex'	
187	'PYDXK'	'PYDXPP'	
188	'PSD181'	'PSSA181'	
189	'PSD160'	'PSSA160'	
190	'PSD141'	'PSSA141'	
191	'PSD140'	'PSSA140'	

192	'PROt2rpp'	'PROt4pp'
193	'PPTHpp'	'PPTtex'
194	'PPPNt2rpp'	'PPPNtex'
195	'CPPPGO'	'PPPGO'
196	'PPCSCT'	'SUCOAS'
197	'PPALtex'	'PPALtpp'
198	'PPAKr'	'PTA2'
199	'PGPP181'	'PGSA181'
200	'PGPP180'	'PGSA180'
201	'PGPP161'	'PGSA161'
202	'PGPP141'	'PGSA141'
203	'PGPP140'	'PGSA140'
204	'PGPP120'	'PGSA120'
205	'PGP181abc	'PGPP181pp'
206	'PGP180abc	'PGPP180pp'
207	'PGP161abc	'PGPP161pp'
208	'PGP160abc	'PGPP160pp'
209	'PGP141abc	'PGPP141pp'
210	'PGP140abc	'PGPP140pp'
211	'PGP120abc	'PGPP120pp'
212	'PDXPP'	'PYDXNK'
213	'OMMBLHX	'OMPHHX3'
214	'NTD5'	'TMDK1'
215	'NTD10pp'	'XMPtex'
216	'NTD10'	'XPPT'
217	'NOVBCNte	'NOVBCNtpp'
218	'NO3t7pp'	'NO3tex'
219	'NO2t2rpp'	'NODOx'
220	'NI2t3pp'	'NI2tpp'
221	'NDPK8'	'RNTR1c2'
222	'NAMNPP'	'NNAM'
223	'NADK'	'NADPPPS'
224	'MN2t3pp'	'MN2tpp'
225	'MLTG5'	'MLTP3'
226	'MLTG4'	'MLTP2'
227	'MLTG3'	'MLTP1'
228	'MINCYCtex	'MINCYCtpp'
229	'MG2t3_2pp	'MG2tpp'
230	'METOX2s'	'METSOXR2'

231	'METOX1s'	'METSOXR1'
232	'MELIBt2pp'	'MELIBt3ipp'
233	'MDDEP2pp	'MLDCP1App'
234	'MDDCP3pp	'MDDCP4pp'
235	'MDDCP1pp	'MDDEP1pp'
236	'MANGLYC	'MANGLYCtex'
237	'MANAO'	'MNNH'
238	'MALTTRab	'MALTTRtexi'
239	'LYSt2pp'	'LYSt3pp'
240	'LPLIPAL2A	'LPLIPAL2ATG180'
241	'LPLIPAL2A	'LPLIPAL2ATG141'
242	'LPLIPAL1G	'PLIPA2G181pp'
243	'LPLIPAL1G	'PLIPA2G180pp'
244	'LPLIPAL1G	'PLIPA2G161pp'
245	'LPLIPAL1G	'PLIPA2G160pp'
246	'LPLIPAL1G	'PLIPA2G141pp'
247	'LPLIPAL1G	'PLIPA2G140pp'
248	'LPLIPAL1G	'PLIPA2G120pp'
249	'LPLIPAL1E	'PLIPA2E181pp'
250	'LPLIPAL1E	'PLIPA2E180pp'
251	'LPLIPAL1E	'PLIPA2E161pp'
252	'LPLIPAL1E	'PLIPA2E160pp'
253	'LPLIPAL1E	'PLIPA2E141pp'
254	'LPLIPAL1E	'PLIPA2E140pp'
255	'LPLIPAL1E	'PLIPA2E120pp'
256	'LPLIPAL1A	'PLIPA2A181pp'
257	'LPLIPAL1A	'PLIPA2A180pp'
258	'LPLIPAL1A	'PLIPA2A161pp'
259	'LPLIPAL1A	'PLIPA2A160pp'
260	'LPLIPAL1A	'PLIPA2A141pp'
261	'LPLIPAL1A	'PLIPA2A140pp'
262	'LPLIPAL1A	'PLIPA2A120pp'
263	'LIPOCT'	'OCTNLL'
264	'LEUabcpp'	'LEUt2rpp'
265	'LCTSt3ipp'	'LCTStpp'
266	'LALDO3'	'LCADi'
267	'LALDO2x'	'LCARR'
268	'Kt2pp'	'Kt3pp'
269	'DMPPS'	'IPDDI'

270	'INSt2pp_cc	'INSt2pp_copy2'
271	'ILEabcpp'	'ILEt2rpp'
272	'IDOND'	'IDOND2'
273	'HYPOE'	'PYDAMK'
274	'HPPPNt2rp	'HPPPNtex'
275	'HOPNTAL'	'OP4ENH'
276	'HKNDDH'	'HPPPNDO'
277	'HISabcpp'	'HISt2rpp'
278	'HEX7'	'XYLI2'
279	'GUAt2pp'	'GUAtpp'
280	'GUACYC'	'PDE4'
281	'GTPHs'	'NTPP11'
282	'GTHRDabc	'GTHRDabcpp'
283	'GSPMDA'	'GSPMDS'
284	'GRXR'	'GTHOr'
285	'GLYOX'	'LGTHL'
286	'GLYCLTt2r	'GLYCLTt4pp'
287	'GLUDy'	'H2Otpp'
288	'GLCtex_co	'GLCtex_copy2'
289	'GLCS1'	'GLGC'
290	'GLCRD'	'GLCRt2rpp'
291	'GDPMNH'	'PMANM'
292	'GARFT'	'GART'
293	'GALUi'	'TRE6PS'
294	'GALURt2rp	'GUI2'
295	'GALCTD'	'GALCTt2rpp'
296	'G6PDA'	'GF6PTA'
297	'G5SD'	'GLU5K'
298	'G5SADs'	'P5CD'
299	'G3PStex'	'GPDDA3pp'
300	'G3PSabcp	'GPDDA3'
301	'FUSAtex'	'FUSAtpp'
302	'FRD3'	'NADH18pp'
303	'FRD2'	'NADH17pp'
304	'FORt2pp'	'FORtppi'
305	'FOMETRi'	'THFAT'
306	'FE3DHBZS	'FE3DHBZStonex'
307	'FBA3'	'PFK_3'
308	'FALDtex'	'FALDtpp'

309	'FACOAL60	'HEXt2rpp'
310	'FACOAE18	'FACOAL181t2pp'
311	'FACOAE18	'FACOAL180t2pp'
312	'FACOAE16	'FACOAL161t2pp'
313	'FACOAE16	'FACOAL160t2pp'
314	'FACOAE14	'FACOAL141t2pp'
315	'FACOAE14	'FACOAL140t2pp'
316	'FACOAE12	'FACOAL120t2pp'
317	'ETHAt2pp'	'GPDDA2pp'
318	'ETHAAL'	'GPDDA2'
319	'EDA'	'EDD'
320	'DTMPtex'	'NTD5pp'
321	'DSERt2pp'	'DSERtex'
322	'DSBDR'	'TDSR1'
323	'DOXRBCN	'DOXRBCNtpp'
324	'DHFR'	'TMDS'
325	'DHBD'	'ICHORT'
326	'DGK1'	'NTD8'
327	'DDGLCNt2	'DDGLCNtex'
328	'DCYTD'	'NTD3'
329	'DAGK141'	'PAPA141'
330	'DAGK120'	'PAPA120'
331	'CYTK1'	'NDPK3'
332	'CYTDt2pp_	'CYTDt2pp_copy2'
333	'CYSabcpp'	'CYStpp'
334	'CYSS'	'SERAT'
335	'CUt3'	'CUtex'
336	'CU2abcpp'	'CU2tpp'
337	'CTBTabcp	'CTBTt2rpp'
338	'CRNCBCT'	'CRNCDH'
339	'COBALT2t	'COBALT2tpp'
340	'CMtex'	'CMtpp'
341	'CMPN'	'CSND'
342	'CLPNH181	'CLPNS181pp'
343	'CLPNH180	'CLPNS180pp'
344	'CLPNH161	'CLPNS161pp'
345	'CLPNH160	'CLPNS160pp'
346	'CLPNH141	'CLPNS141pp'
347	'CLPNH140	'CLPNS140pp'

348	'CLPNH120	'CLPNS120pp'
349	'CITt3pp'	'CITt7pp'
350	'CHLt2pp'	'G3PCabcpp'
351	'CD2t3pp'	'CD2tpp'
352	'CCGS'	'CDGS'
353	'BWCOS'	'WCOS'
354	'ATPHs'	'NTPP9'
355	'ATHRDHr'	'THRA2'
356	'ARGAGMt7	'ARGDCpp'
357	'AP4AH'	'AP4AS'
358	'ALR2'	'ALR4x'
359	'ALAR'	'ALATA_D2'
360	'ALAALAab	'MCTP1Bpp'
361	'ALAALAD'	'ALAALAr'
362	'AGM3PH'	'AM3PA'
363	'ADSL1r'	'ADSS'
364	'ADPRDP'	'NADN'
365	'ADNt2pp_c	'ADNt2pp_copy2'
366	'ADNCYC'	'PDE1'
367	'ADK4'	'NTP10'
368	'ADK3'	'NDPK1'
369	'ACt2rpp'	'ACt4pp'
370	'ACPPAT18	'APH181'
371	'ACPPAT16	'APH161'
372	'ACPPAT16	'APH160'
373	'ACPPAT14	'APH140'
374	'ACPPAT12	'APH120'
375	'ACOATA'	'MACPD'
376	'ACACt2pp'	'ACACtex'
377	'ABUTD'	'PTRCTA'
378	'AAMYLpp'	'MALTHXabcpp'
379	'AACTOOR'	'AOBUTDs'
380	'AACPS9'	'FA80ACPHi'
381	'AACPS8'	'FA100ACPHi'
382	'AACPS6'	'AGPAT180'
383	'3PEPTabcp	'AGMt2pp'
384	'3GMPtex'	'3NTD9pp'
385	'2MAHMP'	'PMPK'
386	'23PDE4pp'	'3NTD4pp'

387	'14GLUCAN	'AAMYL'
388	'12DGR180	'PAPA180pp'
389	'12DGR160	'PAPA160pp'
390	'12DGR141	'PAPA141pp'
391	'12DGR140	'PAPA140pp'
392	'12DGR120	'PAPA120pp'
393	'EX_xan_e'	'XANtex'
394	'EX_val__L_	'VALtex'
395	'EX_uri_e'	'URItex'
396	'EX_ura_e'	'URAtex'
397	'EX_ump_e'	'UMPtex'
398	'EX_thymd_	'THMDtex'
399	'EX_thr__L_	'THRtex'
400	'EX_succ_e	'SUCCtex'
401	'EX_ser__L_	'SERtex'
402	'EX_ptrc_e'	'PTRCtex'
403	'EX_pro__L_	'PROtex'
404	'EX_orn_e'	'ORNtex'
405	'EX_no2_e'	'NO2tex'
406	'EX_nmn_e'	'NMNtex'
407	'EX_na1_e'	'NAtex'
408	'EX_mal__L	'MALtex'
409	'EX_lys__L_	'LYStex'
410	'EX_lipa_e'	'LIPAabctex'
411	'EX_leu__L_	'LEUtex'
412	'EX_ins_e'	'INStex'
413	'EX_inost_e	'INSTtex'
414	'EX_indole_	'INDOLEtex'
415	'EX_ile__L_	'ILEtex'
416	'EX_hxa_e'	'HXAtex'
417	'EX_his__L_	'HIStex'
418	'EX_gua_e'	'GUAtex'
419	'EX_gthrd_e	'GTHRDtex'
420	'EX_glyclt_e	'GLYCLTtex'
421	'EX_glyc3p_	'GLYC3Ptex'
422	'EX_glyc_e'	'GLYCtex'
423	'EX_gly_e'	'GLYtex'
424	'EX_glu__L_	'GLUtex'
425	'EX_glcur1p	'GLCUR1Ptex'

426	'EX_glcn_e'	'GLCNtex'
427	'EX_g3pg_e	'G3PGtex'
428	'EX_g3pe_e	'G3PEtex'
429	'EX_for_e'	'FORtex'
430	'EX_etha_e'	'ETHAtex'
431	'EX_enlipa_	'ENLIPAabctex'
432	'EX_cytd_e'	'CYTDtex'
433	'EX_cys__L	'CYStex'
434	'EX_crn__D	'CRNDtex'
435	'EX_crn_e'	'CRNtex'
436	'EX_colipa_	'COLIPAabctex'
437	'EX_cit_e'	'CITtex'
438	'EX_chtbs_	'CHTBStex'
439	'EX_cgly_e'	'CGLYtex'
440	'EX_cd2_e'	'CD2tex'
441	'EX_cbl1_e'	'CBL1tonex'
442	'EX_asp__L	'ASPtex'
443	'EX_asn__L	'ASNtex'
444	'EX_arg__L	'ARGtex'
445	'EX_arab__	'ARBtex'
446	'EX_anhgm	'ANHGMtex'
447	'EX_alaala_	'ALAALAtex'
448	'EX_ala__L	'ALAtex'
449	'EX_ala__D	'DALAtex'
450	'EX_agm_e'	'AGMtex'
451	'EX_adn_e'	'ADNtex'
452	'EX_ac_e'	'ACtex'
453	'EX_LalaDg	'4PEPTtex'
454	'EX_LalaDg	'3PEPTtex'
455	'EX_4abut_	'ABUTtex'
456	'GK1'	
457	'EX_glc__D_e'	
